# Historical Biogeography of Melanthiaceae: A Case of Out-of-North America Through the Bering Land Bridge

**DOI:** 10.3389/fpls.2019.00396

**Published:** 2019-04-04

**Authors:** Changkyun Kim, Sang-Chul Kim, Joo-Hwan Kim

**Affiliations:** ^1^Department of Life Science, Gachon University, Seongnam, South Korea; ^2^Division of Forest Genetic Resources, National Institute of Forest Science, Suwon, South Korea

**Keywords:** biogeography, bering land bridge, dispersal, melanthiaceae, molecular dating, out-of-North America

## Abstract

Intercontinental floristic disjunction between East Asia and North America in the Northern Hemisphere has received much attention during the past decades, but few studies have focused on the family level. Melanthiaceae, containing 196 species and 17 genera circumscribed in five tribes, is disjunctly distributed in Eurasia and North America. It is one of the foremost models for studying the evolution of biogeographic patterns in this region. Here, we present a fossil-calibrated, molecular phylogeny of Melanthiaceae based on two chloroplast DNA datasets: one dataset includes extensive sampling (94 species representing all 17 genera of Melanthiaceae) of four chloroplast DNA regions (*atpB*, *rbcL*, *matK*, and *ndhF*) and the other includes six species representing all tribes of the family for 78 coding genes of the chloroplast genome. Within this framework, we infer the historical biogeography of Melanthiaceae. Both datasets produce well-resolved phylogenies of Melanthiaceae showing the monophyly of the family and the relationships among the five tribes. Melanthieae is found to be sister to the rest of the tribes of the family and the remaining taxa are divided into two major clades consisting of the Chionographideae + Heloniadeae clade and the Parideae + Xerophylleae clade. The molecular dating and the ancestral area analyses suggest that Melanthiaceae most likely originated in North America with its crown group dated at 92.1 mya in the late Cretaceous. The favored ancestral areas at the crown lineages of tribes are also in North America. In the family, seven independent migrations into East Asia from North America are inferred to have occurred in the Oligocene and the Miocene-Pliocene via historical paleo-land bridge connections. Cooling trends during the Oligocene resulted in the present East Asia-North America disjunct distribution, while the warm period during the middle Miocene and habitat heterogeneity stimulated diversification in East Asia. Our study provides the phylogenetic and biogeographical history of the Melanthiaceae and adds an example of “out of North America” migration in the biogeographic history of the Northern Hemisphere.

## Introduction

The historical biogeography of intercontinental disjunctions between eastern Asia and North America in the Northern Hemisphere has long been of interest to botanists and biogeographers (Donoghue et al., 2001; Wen et al., 2010, 2016). The biogeographic origin of disjunct lineages in the Northern Hemisphere, the timing of this disjunction, and putative migration pathway for many disjunct taxa have been evaluated based on molecular data (Wen et al., 2010; Zhao et al., 2013; Kim et al., 2015, 2017). The evidence available so far indicates a complex history of this disjunction pattern involving exchanges of plants between eastern Asia and North America at different geographical times via paleo-land bridge connections (the North Atlantic land bridge and the Bering land bridge) and long-distance dispersal (Wen et al., 2010; Deng et al., 2015, 2018; Kim et al., 2015). Based on the revision of 98 plant lineages with disjunct distributions between eastern Asia and North America, Wen et al. (2010) found that most temperate lineages originated from eastern Asia and subsequently migrated to North America. Most of these studies have focused at the genus level and woody plants, but few studies have examined the biogeographic pattern of the disjunction in terrestrial herbs at the family level (Deng et al., 2015).

The Melanthiaceae (Liliales) is a family of flowering perennial herbs. This family consists of approximately 196 species in 17 genera and is divided into five tribes, consisting of Heloniadeae, Chionographideae, Xerophylleae, Melanthieae, and Parideae (APG, 2016; Kim et al., 2016). The members of the family are recognized as a monophyletic group by molecular evidence and the morphological features of extrorse anthers and three forms of ovaries (Rudall et al., 2000; Kim et al., 2016). Furthermore, previous studies have provided insights into the phylogenetic relationships among the five tribes in the family (Zomlefer et al., 2001, 2006; Fay et al., 2006). Particularly, a recent phylogenetic study based on plastid DNA sequences confirmed that Melanthieae was sister to the remaining tribes of the family (Kim et al., 2016). Sister relationships between Xerophylleae + Parideae and Chionographideae + Heloniadeae were also supported. Although clear phylogenetic relationships of Melanthiaceae have been recovered, these studies did not examine the biogeographic origin of the family.

**Figure 1:**
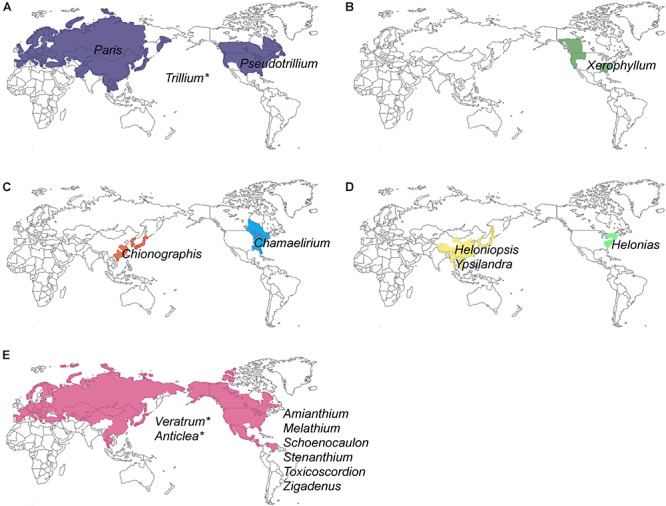
Geographical distribution of 17 genera and five tribes of Melanthiaceae showing disjunctions between Eurasia and North America: **(A)** Parideae; **(B)** Xerophylleae; **(C)** Chionographideae; **(D)** Heloniadeae; **(E)** Melanthieae. An asterisk indicates the genera that were distributed in both Eurasia and North America (see Table 1 for a detailed distribution of genera).

Unlike most families of Liliales, which are found in the Southern Hemisphere (with the exception of the Liliaceae), most species of Melanthiaceae occur in the temperate regions of East Asia and North America (Zomlefer et al., 2001, 2006; Liao et al., 2007; APG, 2016) representing a pattern of the well-known East Asian-North American floristic disjunction. Species of *Paris* L., *Heloniopsis* A.Gray, *Ypsilandra* Franch., and *Chionographis* Maxim. are restricted to East Asia, and species of *Xerophyllum* Michx., *Pseudotrillium* S.B.Farmer, *Helonias* Adans., *Chamaelirium* Willd., *Amianthium* A.Gray, *Melanthium* L., *Stenanthium* (A.Gray) Kunth, *Toxicoscordion* Rydb., and *Zigadenus* Michx. occur only in North America, whereas *Trillium* L., *Veratrum* L., and *Anticlea* Kunth. can be found on both continents; exceptions include *Schoenocaulon* A.Gray, which mainly grows in Mexico and *S. officinale* A.Gray, which expanded its range as far south as South America (Figure 1 and Table 1). Therefore, Melanthiaceae offers an ideal opportunity for studying the biogeography and diversification of an East Asian-North American disjunct group that is presently distributed across the two continents.

**Table 1:** The number of species and distribution of currently recognized genera and tribes of Melanthiaceae.

Tribes	Genera	No. of species	Distribution
Parideae	*Paris*	27	Eurasia
	*Pseudotrillium*	1	western North America
	*Trillium*	51	Eurasia, North America
Xerophylleae	*Xerophyllum*	2	North America
Chionographideae	*Chamaelirium*	1	eastern North America
	*Chionographis*	6	eastern Asia
Heloniadeae	*Helonias*	1	eastern North America
	*Heloniopsis*	6	eastern Asia
	*Ypsilandra*	6	eastern Asia
Melanthieae	*Amianthium*	1	North America
	*Anticlea*	12	Asia, North America
	*Melanthium*	4	North America
	*Schoenocaulon*	26	southern North America to Peru
	*Stenanthium*	3	North America
	*Toxicoscordion*	8	western North America
	*Veratrum*	27	Eurasia, North America
	*Zigadenus*	1	southeastern North America

In order to rigorously determine the mechanisms of intercontinental disjunctions, divergence dates with molecular data are required (Donoghue et al., 2001; Dragon and Barrington, 2009). However, the divergence time of Melanthiaceae is contentious (Vinnersten and Bremer, 2001; Janssen and Bremer, 2004; Mennes et al., 2015). Vinnersten and Bremer (2001) used molecular data to estimate the divergence times for families and major clades within Liliales. The study suggested that the crown node of Melanthiaceae was estimated at 42–54 million years ago (mya) in the Eocene. Based on the analysis using *rbcL* and fossil calibration points, Janssen and Bremer (2004) proposed that Melanthiaceae originated at 97 mya in the late Cretaceous. Recently, Givnish et al. (2016) suggested that the crown age of Melanthiaceae is 84.8 mya in the late Cretaceous. The divergence times of the families within Liliales were also estimated by Mennes et al. (2015), which is similar to the result by Vinnersten and Bremer (2001), although they may be affected by a comparatively limited taxon sampling. Thus, the exact divergence time of Melanthiaceae is required to elucidate its biogeographic history by a broader taxon sampling scheme of the family.

Here, we further infer the historical biogeography of Melanthiaceae by using a fossil calibrated chronogram based on our previous phylogenetic framework (Kim et al., 2016). The ancestral area of Melanthiaceae and subsequent migration routes are interpreted and compared with the molecular dating results to assess the most reasonable explanation for the current distribution of the family.

## Materials and Methods

### Taxon Sampling

Two datasets were designed for this study. The first dataset was based largely on information retrieved from four plastid coding regions (*atpB*, *rbcL*, *matK*, and *ndhF*) from Kim et al. (2016). This dataset included 94 species representing all 17 genera and five tribes of Melanthiaceae and covered the entire extant geographic range of Melanthiaceae *sensu* APG (2016). Twelve families were selected as outgroups of Liliales (Liliaceae, Smilacaceae, Rhipogonaceae, Philesiaceae, Colchicaceae, Luzuriagaceae, Alstroemeriaceae, Petermanniaceae, and Campynemataceae) and Asparagales (Asparagaceae, Amaryllidaceae, and Orchidaceae) based on previous phylogenetic analyses (Patterson and Givnish, 2002; Kim et al., 2013, 2016). For the second molecular dating analyses using 78 coding genes of chloroplast genome, we included six species representing all tribes of Melanthiaceae. We also included five species from Liliales and two species from Asparagales as outgroups. Sources for the taxon names, vouchers, and accession numbers used in this study are listed in Supplementary Table S1.

### Analyses of Phylogeny and Divergence Time

Sequence alignment was performed in MAFFT v.6^1^ (Katoh and Toh, 2008) using default alignment parameters and followed by manual adjustments in BioEdit v.7.2.5 (Hall, 1999). Phylogenetic reconstruction of the four combined plastid DNA regions was performed by Bayesian inference (BI) with MrBayes v.3.1.2 (Ronquist and Huelsenbeck, 2003). Applying the Bayesian Information Criterion (BIC), jmodeltest v.2.1.3 (Darriba et al., 2012) assigned the GTR + I + G model to the sequence data. Four Markov Chain Monte Carlo inferences were run simultaneously and sampled every 1,000 generations for a total of 20 million generations. The first 5,000 (25%) of the sampled trees from each run were discarded as determined by Tracer v.1.6 (Rambaut et al., 2014). The remaining trees were used to construct a 50% majority-rule consensus tree given as posterior probability (PP) to estimate robustness of the BI trees.

A likelihood ratio test provided no support for a molecular clock hypothesis (*P* < 0.05). Thus, we conducted the divergence time analyses using a Bayesian approach implemented in BEAST v.1.8.2 (Drummond et al., 2006; Drummond and Rambaut, 2007). The BEAUti interface was used to generate input files for BEAST, in which a GTR + I + G model (nst = 6, rates = invgamma) for the combined two datasets was applied. A Yule tree prior was selected to calculate the rate of speciation (Gernhard et al., 2008), and uncorrelated and lognormally distributed rate variations were checked via Markov Chain Monte Carlo runs extending for 100 million generations (burn-in 10%) with parameters sampled every 1,000 generations. Tracer v.1.6 (Rambaut et al., 2014) was used to check the effective sample sizes (ESS), which were above 200. The sampled posterior trees were combined using LogCombiner (in BEAST), and a maximum clade credibility tree with mean node heights and 95% highest posterior density (HPD) intervals was generated via TreeAnnotator. The results were visualized with Figtree v.1.4.2 (Rambaut, 2014).

Because there is no reliable fossil assigned to Melanthiaceae, we constrained the ages of the six nodes from outgroups in the first dataset. We used lognormal prior distribution when fossil data was not published as the range in the geologic epoch, but uniformed prior distribution when fossil data was reported as the range. In other cases, we followed the previous studies in setting the constraints as uniformed distribution as follows: (1) the crown node of Asparagales was constrained with a uniform distribution from 124.4 to 125.5 mya, following Magallón and Castillo (2009), who estimated the divergence time of angiosperm orders using the concatenated nucleotide sequences of three chloroplast genes (*atpB*, *rbcL*, *matK*) and two nuclear markers (18S nuclear ribosomal DNA[nrDNA] and 26S nrDNA); (2) the stem node of *Yucca* was calibrated with a uniform distribution from 14.5 to 16.2 mya based on the fossil of *Protoyucca shadishii* represented by permineralized stems from Middle Miocene strata in northwestern Nevada (Tidwell and Parker, 1990; McKain, 2012); (3) the crown node of Liliales was constrained using a uniform distribution from 100 to 130 mya following Givnish et al. (2016), who estimated the divergence time of Liliales using 75 chloroplast genes; (4) the stem node of the *Luzuriaga* clade in Alstroemeriaceae was constrained with a log-normal prior having an offset of 23.2 mya, a mean of 1.0, and a standard deviation (SD) of 0.5. The offset was based on the age of a *Luzuriaga*-like leaf fossil from the Foulden Maar deposits located outside of Otago, New Zealand, which was dated to ca. 23 mya (Conran et al., 2009a); (5) the stem node of *Smilax* was calibrated with a uniform distribution from 37 to 56 mya based on *Smilax*-like fossils dating to the Early Eocene (48.6–55.8 mya; Edelman, 1975; Wilf, 2000) and the Middle Eocene (37.2–48.6 mya; MacGinitie, 1941; Wilde and Frankenhäuser, 1998); and (6) the stem node of the Rhipogonaceae was constrained using a uniform distribution from 51 to 52 mya based on leaf macrofossils of *Rhipogonum* collected in Tasmania dating back to 51–52 mya (Conran et al., 2009b). For the second dataset of 78 coding genes of chloroplast genome, the crown node of Liliales was constrained using a uniform distribution from 100 to 130 mya.

### Reconstruction of the Ancestral Area

The distribution data for species of the Melanthiaceae were compiled from the literature and herbarium specimens (Rydberg, 1903; Dahlgren et al., 1985; Goldblatt, 1995; Zomlefer, 1997; Tamura, 1998; Chen et al., 2000; Utech, 2002). The distribution range of Melanthiaceae was divided into four regions, consisting of (A) North America, (B) East Asia, (C) Europe, and (D) Central America. Ancestral area reconstruction and estimating spatial patterns of geographic diversification within Melanthiaceae were inferred using the Bayesian binary method (BBM) and statistical dispersal-vicariance analysis (S-DIVA) as implemented in RASP v.3.2 (Reconstruct Ancestral State in Phylogenies; Yu et al., 2015). The BBM was run with the fixed state frequencies model (Jukes-Cantor) with equal among site rate variation for two million generations, ten chains each, and two parallel runs. In S-DIVA, the frequencies of an ancestral range at a node in ancestral reconstructions are averaged over all trees. For these analyses, we used 100,000 trees obtained from the BEAST MCMC output of four cpDNA datasets after removing outgroups. The consensus tree used to map the ancestral distribution on each node was obtained with the Compute Condense option in RASP from the stored trees. Because some tribes of Melanthiaceae are widely distributed in the Northern Hemisphere and the present distribution of the tribes does not necessarily represent their ancestral area, the maximum number of ancestral areas was maintained at four.

## Results

### Phylogenetic Relationships of Melanthiaceae

**Figure 2:**
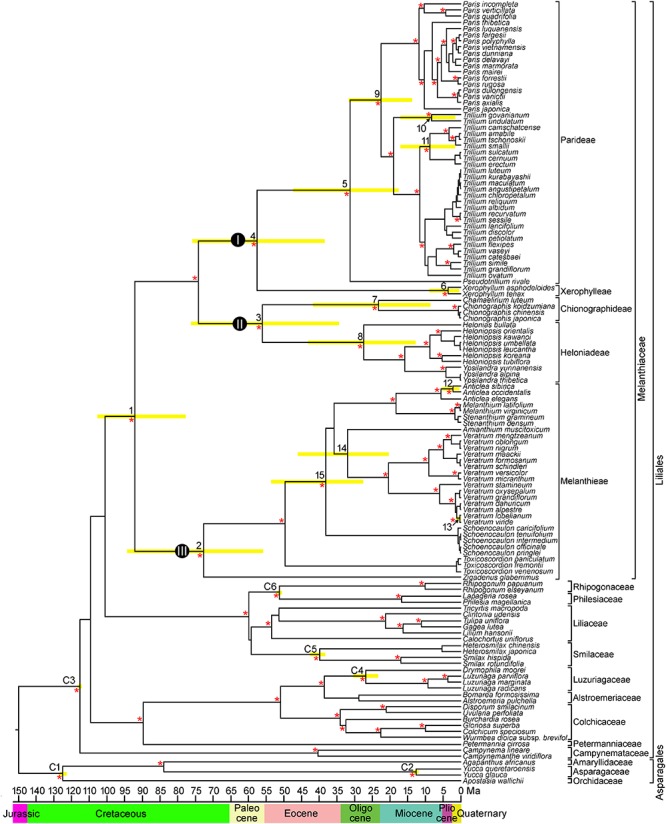
Chronogram showing divergence times estimated in BEAST based on combined four plastid DNA regions (*atpB*, *rbcL*, *matK*, and *ndhF*). Nodes are posterior mean ages with yellow bars representing 95% highest posterior density intervals. Numbers 1–15 indicate nodes of interest (see Table 2 for details). Nodes labeled C1–C6 are calibration points used in the analysis (for more details, see section “Materials and Methods”). Geological epoch is shown below the tree. An asterisk (^∗^) indicates the branches with Bayesian posterior probabilities >0.95.

The combined matrix of the four cpDNA regions (*atpB*, *rbcL*, *matK*, and *ndhF*) from 127 species comprised 6,972 positions, of which 2,633 (37.8%) were variable and 1,977 (28.4%) were potentially parsimony informative. The cp genome dataset included 71,174 aligned nucleotides from 13 species and 78 protein coding genes, of which 9,009 (22.6%) were variable and 7,101 (10.0%) were potentially parsimony informative. The BI phylogram was identical in topology to the maximum clade credibility tree obtained from BEAST (BI phylogram not shown). The BEAST analysis produced a well resolved phylogeny of Melanthiaceae, indicating the monophyly of the family and the relationships among the five tribes and 17 genera (Figure 2). Melanthieae (clade III; PP = 1.00) was found to be a sister to the rest of the family (PP = 1.00), and the remaining taxa were divided into two major clades consisting of Parideae + Xerophylleae (clade I; PP = 1.00) and Chionographideae + Heloniadeae (clade II; PP = 1.00).

In Melanthieae, North American *Zigadenus* was the first branching taxon and *Toxicoscordion* formed the subsequent branch as a sister of the remainder of the tribe (PP = 1.00), with Central American *Schoenocaulon* sister to the remaining genera. *Amianthium* was a sister to *Veratrum*, but with low support (PP = 0.78). *Anticlea* was sister to *Stenanthium*+ *Melanthium* (PP = 1.00). Within *Anticlea*, *A. sibirica* from East Asia was nested showing a close relationship to North American species, *A. occidentalis* and *A. elegans* (PP = 1.00). In Chionographideae, North American *Chamaelirium* formed a clade with *Chionographis* of East Asia (PP = 1.00). Among the Heloniadeae, North American *Helonias* was sister to East Asian *Ypsilandra* and *Heloniopsis* (PP = 1.00). In Parideae, North American *Pseudotrillium* was sister to the remainder of the tribe (PP = 1.00). In *Trillium*, *T. govanianum*, and *T. undulatum* formed the first-branching clade (PP = 1.00) and the *T. cernuum*-*T. erectum* clade from North America was sister to the *T. tschonoskii*-*T. camschatcense* clade (PP = 1.00).

### Molecular Divergence Time Estimates

Mean divergence age estimates and 95% HPDs for nodes of interest from the BEAST analysis based on four cpDNA dataset are presented in Figure 2 and Table 2. The most recent common ancestor of Melanthiaceae (node 1) was estimated to be 92.1 mya with a 95% HPD range of 77.7–105.2 mya in the Cretaceous. The initial diversification of the Melanthiaceae based on the combined 78 coding DNA sequences of chloroplast genome (85.8 mya, 95% HPD = 76.0–103.2 mya; Supplementary Figure S1) generated similar to that from the four combined cpDNA regions. The ages of the nodes of the tribes were estimated to be from the Pliocene [Xerophylleae (node 6), 3.6 mya, 95% HPD = 0.4–9.1 mya] to the late Cretaceous [Melanthieae (node 2), 73.0 mya, 95% HPD = 55.6–94.5 mya]. The mean ages of the crown nodes with disjunction between Eurasia and North America at intergeneric level (nodes 7–9, 14 in Figure 2 and Table 2) cover a range from 22.5 to 32.1 mya (early Miocene–late Oligocene) and from 0.5 to 8.6 mya (Pleistocene–late Miocene) at the infrageneric level (nodes 10–13).

**Table 2:** The estimated age and reconstructed ancestral area of major nodes of Melanthiaceae.

Node^a^	Description	Age estimate^b^		Ancestral area reconstruction^c^	
		Mean (mya)	95% HPD (mya)	BBM (%)	S-DIVA (%)
1	Melanthiaceae	92.1 (85.8)	77.7–105.2 (76.0–103.2)	A (100)	A (100)
2	Melanthieae (clade III)	73.0	55.6–94.5	A (100)	A (100)
3	Chionographideae + Heloniadeae (clade II)	55.8 (54.2)	34.3–76.3 (33.1–74.8)	A (97)	A (100)
4	Parideae + Xerophylleae (clade I)	57.5 (63.6)	38.5–75.7 (46.7–79.7)	A (100)	A (100)
5	Parideae	31.2	18.1–47.4	A (99)	A (100)
6	Xerophylleae	3.6	0.4–9.1	A (100)	A (100)
7	Chionographideae	23.5	8.7–41.6	A (95)	AB (100)
8	Heloniadeae	27.3	13.1–43.1	A (92)	AB (100)
9	*Paris* + *Trillium*	22.5	14.2–31.4	A (92)	AB (100)
10	*Trillium govanianum* + *T. undulatum*	8.2	1.3–17.1	A (95)	AB (100)
11	*Trillium camschatcense*-*T. smalii* + *T. sulcatum*-*T. erectum*	8.6	2.9–15.9	A (97)	AB (100)
12	*Anticlea sibirica* + *A. occidentalis*	2.2	0.3–5.2	A (97)	AB (100)
13	*Veratrum lobelianum* + *V. viride*	0.5	0.005–1.4	B (52), C (19), A (16)	AC (100)
14	*Amianthium* + *Veratrum*	32.1	20.3–45.8	A (96)	AB (100)
15	*Anticlea-Veratrum* + *Schoenocaulon*	38.2	27.1–54.0	A (96)	AD (100)

^a^*Node numbers and biogeographic codes correspond to those in Figure 2, 3. ^*b*^Age estimates of interest nodes based on 78 coding genes of chloroplast genomes are in parentheses. ^*c*^Ancestral areas for each node are represented with >10%*.

### Ancestral Area Reconstruction

**Figure 3:**
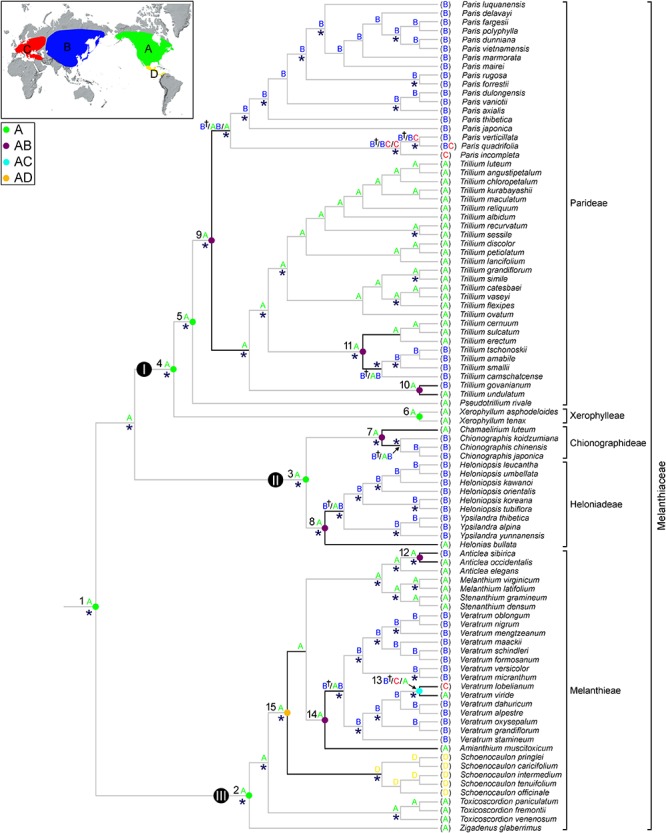
Summary of the Bayesian binary method (BBM) and the S-DIVA models of ancestral area reconstruction in Melanthiaceae based on a reduced BEAST combined-gene chronogram. The results of BBM reconstruction are indicated above the branches. The letter of regions for each node is indicated on the map. S-DIVA ancestral area reconstructions are shown by circles at the nodes of interest (see Table 2 for details). Color key for ancestral reconstruction at nodes of interest obtained from S-DIVA analysis is provided in the figure. The clades (I–III) and numbers (1–15) correspond to those in Figure 2 (see Table 2 for details). An asterisk (^∗^) indicates the branches with Bayesian posterior probabilities >0.95. A dagger (†) indicates the most probable ancestral area for biogeographic regions are shown on the map: A, North America; B, East Asia; C, Europe; D, Central America.

The ancestral distributions inferred from BBM and S-DIVA for internal nodes in Melanthiaceae are shown in Figure 3 and Table 2. Both analyses suggest North America (A) as the ancestral area for Melanthiaceae (node 1), Melanthieae (node 2), Parideae (node 5), and Xerophylleae (node 6). The BBM reconstruction indicates North America (A), followed by East Asia, as the most probable ancestral area for Chionographideae (node 7) and Heloniadeae (node 8), whereas the S-DIVA reconstruction for these nodes are North America + East Asia (AB). Similar results were obtained for the nodes showing disjunction between East Asia and North America. BBM suggests North America (A) as the most probable ancestral area at nodes 9 to 14, except the crown clade of *V. lobelianum*-*V. viride* (node 13, marginal probability = 52%) originated from East Asia, whereas S-DIVA indicates North America + East Asia (AB) or North America + Europe (AC) as the ancestral range at these nodes. Dispersal into Central America from North America was observed in *Schoenocaulon* (node 15), which diverged at 38.2 mya (95% HPD = 27.1–54.0 mya) in the Eocene.

## Discussion

### Divergence Time and Biogeographical Origin of Melanthiaceae and Tribes

The BEAST analysis provides a robust phylogeny of Melanthiaceae that is congruent with our previous phylogenetic results (Kim et al., 2016) and suggests an origin of the family in the late Cretaceous (Figure 2 and Table 2). This result is reinforced by the time estimation based on 78 coding regions of chloroplast genome (Supplementary Figure S1 and Table 2). Our estimation of the crown age of Melanthiaceae is similar to those obtained in previous studies (85–97 mya; Janssen and Bremer, 2004; Anderson and Janssen, 2009; Hertweck et al., 2015; Givnish et al., 2016) or >30 mya earlier than that suggested by Vinnersten and Bremer (2001) and Mennes et al. (2015). Differences from the latter studies are likely to be the result of different calibration points or different tree topologies used, coupled with the use of less comprehensive gene or taxon sampling [e.g., the use of a single *rbcL* sequence by Vinnersten and Bremer (2001)]. We believe that our dataset—which used a broader taxon sampling and the ages of six outgroup fossils—provides the most reliable estimate of the divergence time for the family today.

Our BBM and S-DIVA analyses suggested North America as the ancestral area of Melanthiaceae (Figure 3). Although there is no accurate study on North American fossils of Melanthiaceae that could reveal interesting aspects of the origin and the distribution pattern of the family, North America is suggested to have played an important role in the early evolution of Melanthiaceae. The favored ancestral areas at the crown lineages of tribes were also North America, which suggested an early origin and differentiation of the family in this area. According to Vinnersten and Bremer (2001) and Givnish et al. (2016), the ancestor of the Liliales occurred in the Southern Hemisphere with the descent clade, consisting of the Melanthiaceae, Smilaceae, and Liliaceae, spreading into the Northern Hemisphere during the Cretaceous. At this time, the climate was similar to that of the present-day tropics, characterized by high rates of precipitation and warm temperatures (Wolfe and Upchurch, 1987). The ancestor of the family then expanded its geographic range to the north and subsequently adapted to the climate and the ecological factors of the northern Hemisphere.

In Melanthieae, the divergence of *Zigadenus* began at 73.0 mya (95% HPD = 55.6–94.5 mya) in the late Cretaceous (Figure 2 and Table 2), and *Toxicoscordion* diverged in the Eocene (49.6 mya, 95% HPD = 36.4–65.8 mya). *Schoenocaulon* was found to have spread from North America to Central America at 38.2 mya (95% HPD = 27.1–54.0 mya) in the Eocene and then the genus differentiated at 1.1 mya (95% HPD = 0.09–2.6 mya) in South America (Figure 2). The divergence of *Schoenocaulon* from its North American ancestor broadly coincides with a possible biological connection between North and South America around the Eocene–Oligocene interface (Wolfe, 1975; Nie et al., 2012). On the other hand, it is also possible that *Schoenocaulon* spread to South America as a result of human interference (Frame, 2001; Zomlefer et al., 2006). *Schoenocaulon officinale*, originated in Mexico, is the primary source of the medicinal sabadilla (alkaloids deriving from the seeds), used as a disinfectant and topical pesticide (Zomlefer, 1997). This species was an important drug plant for Native Americans of Mexico and was later cultivated by European settlers for eradicating lice in the 16th century. Plants were even grown over a vast area ranging from Mexico to Venezuela for export to Europe during the World War I for pediculosis (Frame, 1989, 1990). Further studies should be implemented to evaluate the biogeographic history of this genus with more extensive taxon sampling.

Unlike the genera of Melanthieae which originated in North America, our biogeographical analysis points to East Asia as the center of origin of *Veratrum*, the sister group of North American *Amianthium*, and subsequent spread to North America and Europe (Figure 3). This pattern can be supported by the view of Wulff (1943), who suggested that higher species diversity would be found in ancestral areas. Twenty-seven species of *Veratrum* are distributed across Eurasia and North America (Zomlefer et al., 2003). Twenty species occur in East Asia, three in Europe and Central Asia, and four in North America. With the richest species diversity, it could be suggested that the center of diversity of the genus is located in East Asia. Liao et al. (2007) also similarly indicated East Asia as the ancestral area of *Veratrum* using the nrITS sequence data.

In the remaining tribes, the Xerophylleae + Parideae clade and the Chionographideae + Heloniadeae clade diverged at 55.8 mya (95% HPD = 34.3–76.3 mya) and 57.5 mya (95% HPD = 38.5–75.7 mya) in the early Eocene, respectively (Table 2). The separation of Xerophylleae from Parideae in the early Eocene and its recent diversification in the Pliocene could suggest the maintenance of ancestral traits in Xerophylleae for long periods. Morphologically, members of Xerophylleae are more similar to Melanthieae than Parideae. That is, both Xerophylleae and Melanthieae share bulb-shaped rhizomes, calcium oxalate crystals in the raphids and styloids, the production veratrum alkaloids, and sensitivity to particular uredinales (Goldblatt, 1995; Zomlefer, 1997; Kim et al., 2016). Our result relating to the evolutionary scenario for Xerophylleae having small genome sizes (1*C*-value = 2.96–3.46 pg; Pellicer et al., 2014) is also supported by the view of Pellicer et al. (2014) who suggested that the ancestor of Melanthiaceae had a relatively small genome and underwent only one episode of dramatic DNA accumulation in Parideae (1*C*-value = 27.51–74.79 pg; Pellicer et al., 2014).

The tribe Parideae, comprising *Pseudotrillium*, *Paris*, and *Trillium*, diversified at 31.2 mya (95% HPD = 18.1–47.4 mya) in the Oligocene. The monotypic genus *Pseudotrillium* is an early divergent group, making North America a plausible ancestral area for this tribe. Ancestral area reconstruction with BBM and S-DIVA based on our phylogeny supports this view (Figure 3). Farmer (2006) also suggested that this tribe originated in the Pacific Northwest region of North America. Given that *Paris* and *Trillium* are sister groups occupying largely different regions (Eurasia in *Paris* and Asia and North America in *Trillium*), it is difficult to assess their biogeographic origin using a taxon-area cladogram. However, several patterns are inferred from our molecular dating and ancestral area reconstruction. The BBM analysis indicates that North America was the area of origin for the *Paris*-*Trillium* clade with high marginal probability (92%) at 22.5 mya (95% HPD = 14.2–31.4 mya) during the Oligocene/Miocene interface (Figure 2, 3). The North American origin for the *Paris*-*Trillium* species is also supported by the distribution pattern of chromosome numbers. The polyploid species of *Paris* and *Trillium* occur only in the Old World, whereas most diploid species are found in North America (Haga, 1942; Sparrow and Pond, 1950; Kozuka et al., 1964; Li, 1998; Farmer, 2006). The most recent common ancestor of *Paris* probably originated in East Asia and subsequently spread to Europe during the Miocene. In contrast, the ancestor of *Trillium* appeared in North America, followed by the diversification of species that occurred in North America with two dispersals to East Asia. Thus, our results suggest that *Trillium* has a potentially different biogeographic history with *Paris* after the divergence of the common ancestor of both genera.

The remaining tribes, Chionographideae and Heloniadeae, appear to have originated in North America, at 23.5 mya in the early Miocene and 27.3 mya in the late Oligocene, respectively (Figure 3 and Table 2), with the ancestors of *Chionographis* and *Heloniopsis- Ypsilandra* (China, Japan, and Korea) dispersing into East Asia. Thus, both tribes show an intercontinental disjunction between East Asia and North America at the intergeneric level (see details below).

### Intercontinental Disjunctions Between East Asia and North America in Melanthiaceae

East Asia usually has a higher level of species richness and endemism, and it has been suggested to be the ancestral area for many East Asian-North American disjunct groups (Qian and Ricklefs, 1999; Donoghue and Smith, 2004). For example, Donoghue and Smith (2004) found a predominance of directionality in plant groups from East Asia to North America. Of the 51 lineages they analyzed, 20 (39%) showed directionality from East Asia to North America, only two lineages (4%) were inferred to have dispersed from North America to East Asia, whereas the directionality for 29 lineages (57%) was uncertain. However, our biogeographic reconstructions suggest a North American origin for the groups showing intercontinental disjunction between East Asia and North America (Figure 3 and Table 2). Wen et al. (2010) also found many more lineages with North American origins and subsequent migrations into East Asia. Twenty-nine of the 98 total examined plant lineages (30%) migrated from North America to East Asia. Therefore, we suggest that East Asia has been over-emphasized as an ancestral area for taxa disjunctly distributed in both regions.

At the intergeneric level of Melanthiaceae, our molecular dating analyses show that East Asian-North American disjunct lineages have an estimated divergence time between 23.5 (*Chamaelirium*-*Chionographis*; node 7 in Figure 2 and Table 2) and 32.1 mya (*Amianthium*-*Veratrum*; node 14) in the Oligocene. The BBM analysis indicated that the East Asian genera most likely originated in North America (nodes 7–9, 14). During the Cenozoic Epochs, two routes for plant dispersal between the Old World and New World were the North Atlantic land bridge and the Bering land bridge (Tiffney, 1985a; Wen et al., 2010, 2016). These routes have played significant roles in the formation of the modern flora of the Northern Hemisphere, but they occurred in different geographic times. The North Atlantic land bridge, which is a connection between North America and North-eastern Europe via Greenland, has been viewed as a crucial route for the spread of thermophilic taxa of the boreotropical flora during the Paleocene and the early Eocene (Wolfe, 1975; Tiffney, 1985b; Tiffney and Manchester, 2001). In contrast, the Bering land bridge is assumed to have existed from the Paleocene to the Miocene (Tiffney, 1985a; Gladenkov et al., 2002). Therefore, we suggest that the genera that disjunctly distributed in East Asia and North America most likely migrated from North America to East Asia via the Bering land bridge in the Oligocene. The Bering land bridge supported exchanges of temperate plants, but it was ultimately disrupted by a sharp decrease in average temperature from the Oligocene to the present (Zachos et al., 2001). The climate cooling during the Oligocene could have prohibited the interchange between East Asian and North American plants. It also could have facilitated the southward movement in each region and the warm period during the middle Miocene and habitat heterogeneity stimulated the diversification in East Asia, resulting in intercontinental disjunction between East Asia and North America.

Four cases (nodes 10–13 in Figure 3) of disjunct distribution between Eurasia and North America were detected within the genera of Melanthiaceae ranging from 0.5 (*V. lobelianum*-*V. viride*; node 13) to 8.6 mya (*T. camschatcense*-*T. smalii* + *T. sulcatum*-*T. erectum*; node 11). The common ancestors of all Eurasian species came from North America, except for *V. lobelianum*-*V. viride*, which originated from East Asia, suggesting that the divergence times of North American origin are profoundly older than those from East Asia. According to Chen et al. (2017)’s review on evolutionary history of East Asian flora, this region has been assumed as a survival center in which many relict taxa persisted until more recent times due to less influence of Quaternary glaciations and a lower extinction rate during that time. Several recent studies of other taxa in angiosperm based on molecular data have showed examples of an “out-of-North America” origin [e.g., *Smilax hispida* group (Zhao et al., 2013); *Philadelphus* (Guo et al., 2013); Theaceae (Li et al., 2013)].

## Conclusion

In conclusion, our study provides an evolutionary framework to evaluate the biogeographic history of Melanthiaceae. Our molecular phylogeny strongly supports the monophyly of the family and the relationships among the five tribes. We suggest that North America is a major center of origin for taxa currently occurring in East Asia and elsewhere in the Northern Hemisphere. The Bering land bridge may have played a role in the migration in Melanthiaceae from North America to the Old World. Cooling trends during the Oligocene resulted in the present East Asia-North America disjunction. Our study adds an example of “out-of-North America” migration in the biogeographic history of the northern Hemisphere.

## Author Contributions

J-HK conceived and designed the experiments and revised the draft. J-HK and S-CK collected the plant materials. CK, S-CK, and J-HK performed the experiments and analyzed the data. CK and S-CK wrote the draft. All authors agreed on the contents of the manuscript.

## Conflict of Interest Statement

The authors declare that the research was conducted in the absence of any commercial or financial relationships that could be construed as a potential conflict of interest.

## Footnotes

1 http://www.genome.jp/tools/mafft/

## Supplementary Material

FIGURE S1 Chronogram showing the divergence times estimated in BEAST based on the combined 78 coding regions of cp genome. The divergence times of interest nodes are shown near each node. Yellow bars represent 95% highest posterior density for the estimated mean dates. Nodes labeled C3 are the calibration points used in the analysis (for more details, see Materials and Methods).

TABLE S1 Species, voucher with collection locality and GenBank accession number for taxa included in this study.
