# Supplementary figures and images for: Historical Biogeography of Melanthiaceae: A Case of Out-of-North America Through the Bering Land Bridge
Source: Front Plant Sci. 2019 Apr 4;10:396. doi: 10.3389/fpls.2019.00396 (PMC6458295; doi:10.3389/fpls.2019.00396)

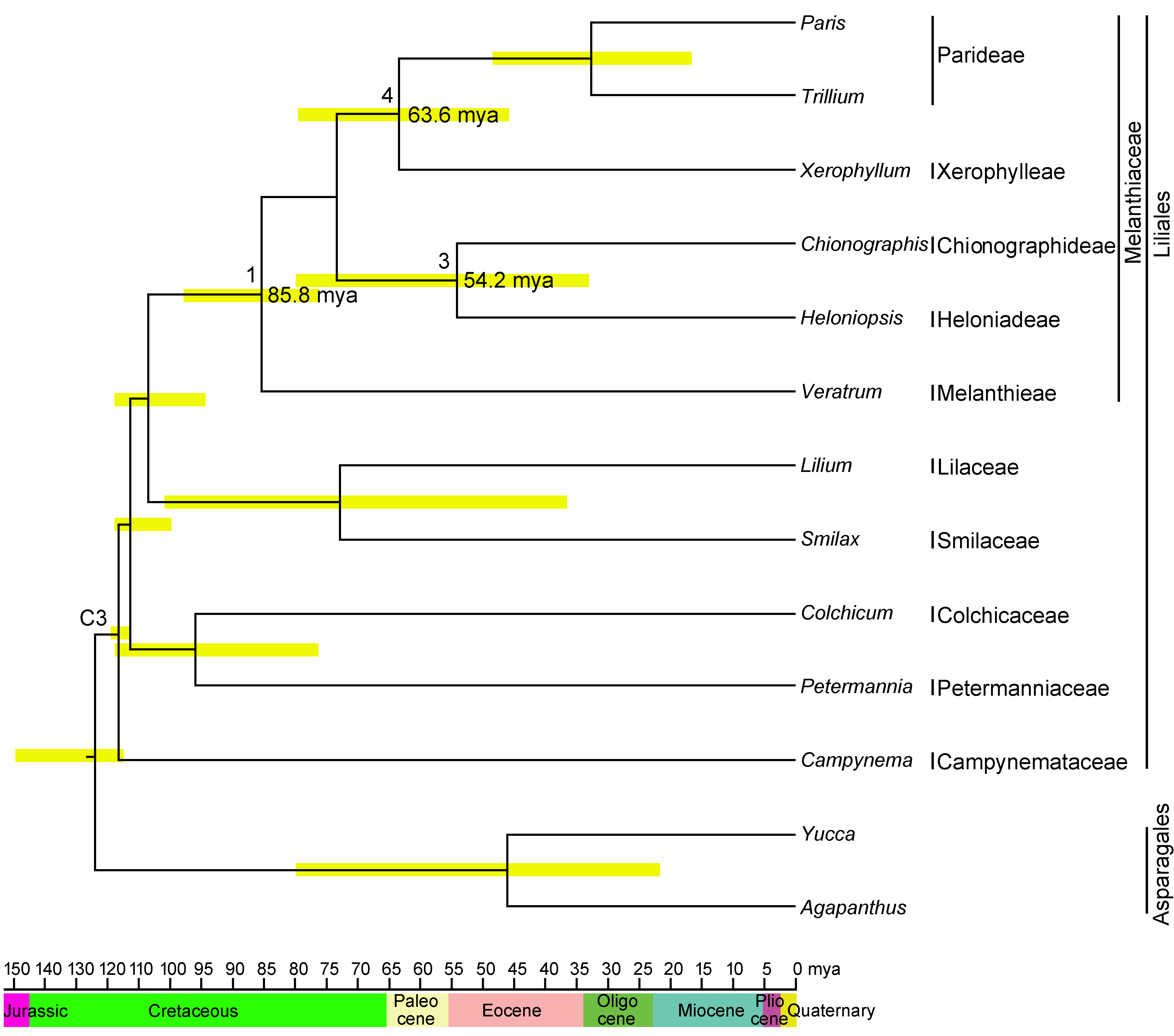

Supplement: FIGURE S1 — Chronogram showing the divergence times estimated in BEAST based on the combined 78 coding regions of cp genome. The divergence times of interest nodes are shown near each node. Yellow bars represent 95% highest posterior density for the estimated mean dates. Nodes labeled C3 are the calibration points used in the analysis (for more details, see Materials and Methods). [file Image_1.TIF]
