# Supplementary material for: Historical Biogeography of Melanthiaceae: A Case of Out-of-North America Through the Bering Land Bridge
Source: Front Plant Sci. 2019 Apr 4;10:396. doi: 10.3389/fpls.2019.00396 (PMC6458295; doi:10.3389/fpls.2019.00396)
Supplement: TABLE S1 — Species, voucher with collection locality and GenBank accession number for taxa included in this study. [file Table_1.DOCX]

**TABLE S1.** Species, voucher with collection locality and GenBank accession number for taxa included in this study.

| **Family/Tribe** | **Species Name** | **BBM**  **scoring** | **Voucher** | **Herbarium** | ***atp*B** | ***rbc*L** | ***mat*K** | ***ndh*F** | ***Cp genome*** |
| --- | --- | --- | --- | --- | --- | --- | --- | --- | --- |
| **Malathiaceae** |  |  |  |  |  |  |  |  | - |
| Chionographideae | *Chamaelirium luteum* (L.) A.Gray | A | H.Koyama 6257 | TI | AF209561 | AJ276347 | AB040196 | AY225006 | - |
| Chionographideae | *Chionographis chinensis* K. Krause | B | W.T. Tsang 24930 | KUN | - | KM242926 | KM242768 | KM242838 | - |
| Chionographideae | *Chionographis japonica* (Willd.) Maxim. | B | D.K.Kim 04-115 | GCU | JX903932 | JN417464 | JN417374 | JX903513 | KF951065 |
| Chionographideae | *Chionographis koidzumiana* Ohwi | B | H.I.Keda & T.Yahara s.n. | KUN | - | KM242927 | KM242769 | KM242839 | - |
| Heloniadeae | *Helonias bullata* L. | A | Chase 41431 | K | KM242696 | KM242928 | KM242770 | KM242842 | - |
| Heloniadeae | *Heloniopsis kawanoi* (Koidz.) Honda | B | Chase 38707 | K | KM242697 | KM242929 | KM242771 | KM242843 | - |
| Heloniadeae | *Heloniopsis koreana* Fuse, N.S.Lee & M.N.Tamura | B | J.K.Hong 056 | GCU | KM242698 | JN417465 | JN417375 | KM242844 | - |
| Heloniadeae | *Heloniopsis leucantha* (Koidz.) Honda | B | H.Setoguchi JP2701 | TI | - | KM242930 | KM242772 | KM242845 | - |
| Heloniadeae | *Heloniopsis orientalis* (Thunb.) Tanaka | B | Chase 445 | K | KM242699 | KM242931 | KM242773 | KM242846 | - |
| Heloniadeae | *Heloniopsis tubiflora* Fuse, N.S.Lee & M.N.Tamura | B | J.K.Hong 046 | GCU | KM242701 | JN417466 | JN417376 | KM242849 | NC027159 |
| Heloniadeae | *Heloniopsis umbellata* Baker | B | Chase 38708 | K | KM242702 | KM242934 | KM242776 | KM242850 | - |
| Heloniadeae | *Ypsilandra alpina* F.T.Wang & Tang | B | KUN 0773902 | KUN | KM242765 | KM242999 | - | - | - |
| Heloniadeae | *Ypsilandra thibetica* Franch. | B | E.H.Wilson 4676 | K | KM242766 | KM243000 | KR233275 | - | - |
| Heloniadeae | *Ypsilandra yunnanensis* W.W.Sm. & Jeffrey | B | T.T.Yu 19036 | KUN | KM242767 | KM243001 | - | - | - |
| Melanthieae | *Amianthium muscitoxicum* (Walter) A.Gray | A | ND |  | AJ417582 | AJ417895 | FR832722^1^ | - | - |
| Melanthieae | *Anticlea elegans* (Pursh) Rydb. | A | Chase & Fay 14583 | K | KM242692 | JN417468 | JN417378 | KM242835 | - |
| Melanthieae | *Anticlea occidentalis* (A.Gray) Zomlefer & Judd | A | Chase 522 | K | KM242693 | JN417484 | JN417393 | KM242836 | - |
| Melanthieae | *Anticlea sibirica* (L.) Kunth | B | KH801023 | GCU | KM242694 | JN417481 | JN417390 | KM242837 | - |
| Melanthieae | *Melanthium latifolium* Desr. | A | ND |  | - | JN417482 | JN417391 | - | - |
| Melanthieae | *Melanthium virginicum* L. | A | Chase 1024 | K | KM242703 | KM242935 | KM242777 | KM242851 | - |
| Melanthieae | *Schoenocaulon caricifolium* (Schltdl.) A.Gray | D | Chase 1852 | K | KM242725 | JN417483 | JN417392 | KM242872 | - |
| Melanthieae | *Schoenocaulon intermedium* Baker | D | C.G.Pringle 8356 | NY | - | KM242953 | KM242794 | KM242873 | - |
| Melanthieae | *Schoenocaulon officinale* A.Gray | D | B.Dorr & Diaz 7247 | K | - | KM242954 | KM242795 | KM242874 | - |
| Melanthieae | *Schoenocaulon pringlei* Greenm. | D | C.G.Pringle 6415 | NY | - | KM242955 | KM242796 | KM242875 | - |
| Melanthieae | *Schoenocaulon tenuifolium* B.L.Rob. & Greenm. | D | C.G.Pringle 6653 | NY | - | KM242956 | KM242797 | KM242876 | - |
| Melanthieae | *Stenanthium densum* (Desr.) Zomlefer & Judd 2 | A | J.F.Smith & al. 2262 | US | KR233267 | KR233277 | KR233270 | - | - |
| Melanthieae | *Stenanthium gramineum* (Ker Gawl.) Morong 2 | A | M.T.Strong 3919 | US | KR233268 | KR233278 | KR233271 | - | - |
| Melanthieae | *Toxicoscordion fremontii* (Torr.) Rydb. | A | Chase 16319 | K | KM242726 | KM242959 | KM242798 | KM242877 | - |
| Melanthieae | *Toxicoscordion paniculatum* (Nutt.) Rydb. | A | V.D.Depaepe 7 | NSW | KM242727 | JN417480 | JN417389 | KM242878 | - |
| Melanthieae | *Toxicoscordion venenosum* (S.Watson) Rydb. | A | Van der Werff 12922 | K | KM242728 | KM242960 | - | KM242879 | - |
| Melanthieae | *Veratrum alpestre* Nakai | B | Kyoto 6931 | KYO | - | KM242984 | KM242821 | KM242906 | - |
| Melanthieae | *Veratrum dahuricum* (Turcz.) O.Loes. | B | PE 315978 | PE | - | KM242985 | KM242822 | KM242907 | - |
| Melanthieae | *Veratrum formosanum* O.Loes. | B | Y.Ando & al. 142 | HAST | - | KM242986 | KM242823 | KM242908 | - |
| Melanthieae | *Veratrum grandiflorum* (Maxim. ex Miq.) O.Loes. | B | KUN 0303565 | KUN | KM242755 | KM242987 | KM242824 | KM242909 | - |
| Melanthieae | *Veratrum lobelianum* Bernh. | C | Chase 19618 | K | KM242756 | KM242988 | KM242825 | KM242910 | - |
| Melanthieae | *Veratrum maackii* Regel | B | S.T.Lee s.n. | GCU | KM242758 | KM242989 | KM242826 | KM242912 | - |
| Melanthieae | *Veratrum mengtzeanum* O.Loes. | B | KUN 0303607 | KUN | - | KM242991 | KM242828 | KM242914 | - |
| Melanthieae | *Veratrum micranthum* F.T.Wang & Tang | B | KUN 0303613 | KUN | - | KM242992 | KM242829 | KM242915 | - |
| Melanthieae | *Veratrum nigrum* L. | B | KUN 0303642 | KUN | KM242760 | KM242993 | KM242830 | KM242916 | - |
| Melanthieae | *Veratrum oblongum* O.Loes. | B | T.P.Wang s.n. | PE | - | KM242994 | KM242831 | KM242917 | - |
| Melanthieae | *Veratrum oxysepalum* Turcz. | B | J.K.Hong 015 | GCU | KM242761 | JN417478 | JN417387 | KM242918 | - |
| Melanthieae | *Veratrum schindleri* O.Loes. | B | KUN 0303650 | KUN | - | KM242995 | KM242832 | KM242919 | - |
| Melanthieae | *Veratrum patulum* O. Loes. |  | ND |  | - | - | - | - | NC022715 |
| Melanthieae | *Veratrum stamineum* Maxim. | B | Norita s.n. | TI | - | KM242996 | KR233274 | KM242920 | - |
| Melanthieae | *Veratrum versicolor* Nakai | B | D.H.Lee & M.S.Kim s.n. | GCU | KM242762 | JN417479 | JN417388 | KM242921 | - |
| Melanthieae | *Veratrum viride* Aiton | A | Chase 551 | K | KM242763 | KM242997 | KM242833 | KM242922 | - |
| Melanthieae | *Zigadenus glaberrimus* Michx. | A | M.T.Strong 3925 | US | KR233266 | KR233276 | KR233269 | - | - |
| Parideae | *Paris axialis* H.Li | B | S.W.Lee s.n. | KUN | KM242704 | JN417469 | JN417379 | KM242852 | - |
| Parideae | *Paris delavayi* Franch. | B | KUN 0302034 | KUN | KM242705 | KM242936 | KM242778 | KM242853 | - |
| Parideae | *Paris dulongensis* H.Li & Kurita | B | KUN 0301542 | KUN | KM242706 | KM242937 | KM242779 | KM242854 | - |
| Parideae | *Paris dunniana* H.Lév. | B | S.W.Lee s.n. | KUN | KM242707 | KM242938 | KM242780 | KM242855 | - |
| Parideae | *Paris fargesii* Franch. | B | S.C.Kim, 001 | GCU | KM242708 | KM242939 | KR233272 | KM242856 | - |
| Parideae | *Paris forrestii* (Takht.) H.Li | B | KUN 0302514 | KUN | KM242709 | KM242940 | KM242781 | KM242857 | - |
| Parideae | *Paris incompleta* M.Bieb. | C | N. Lachashvili & M. Khutsishvili s.n | NY | KM242710 | JF942774 | AB018832 | - | - |
| Parideae | *Paris japonica* (Franch. & Sav.) Franch. | B | Chase 29052 | K | KM242711 | KM242941 | KM242782 | KM242858 | - |
| Parideae | *Paris luquanensis* H.Li | B | S.C.Kim, 007 | GCU | KM242712 | KM242942 | KM242783 | KM242859 | - |
| Parideae | *Paris mairei* H.Lév. | B | Chase 17511 | K | KM242713 | KM242943 | KM242784 | KM242860 | - |
| Parideae | *Paris marmorata* Stearn | B | ND |  | - | JF942785 | JF954904 | - | - |
| Parideae | *Paris polyphylla* Sm. | B | KUN 0301753 | KUN | KM242715 | KM242945 | KM242786 | KM242862 | - |
| Parideae | *Paris quadrifolia* L. | BC | J.H.Kim *& al.* 20090905-025 | GCU | KM242718 | JN417472 | JN417382 | KM242865 | - |
| Parideae | *Paris rugosa* H.Li & Kurita | B | KUN 0302215 | KUN | KM242719 | KM242948 | KM242789 | KM242866 | - |
| Parideae | *Paris thibetica* Franch. | B | Chase 487 | K | KM242720 | KM242949 | KM242790 | KM242867 | - |
| Parideae | *Paris vaniotii* H.Lév. | B | KUN 0302439 | KUN | KM242721 | KM242950 | KM242791 | KM242868 | - |
| Parideae | *Paris verticillata* M.Bieb. | B | J.K.Hong 011 | GCU | KM242722 | JN417471 | JN417381 | KM242869 | NC024560 |
| Parideae | *Paris vietnamensis* (Takht.) H.Li | B | S.C.Kim, 004 | GCU | KM242723 | KM242951 | KM242792 | KM242870 | - |
| Parideae | *Pseudotrillium rivale* (S.Watson) S.B.Farmer | A | Chase 18990 | K | KM242724 | KM242952 | KM242793 | KM242871 | - |
| Parideae | *Trillium albidum* J.D.Freeman | A | F.H.Utech 84019 | TI | KM242730 | KM242961 | KM242799 | KM242881 | - |
| Parideae | *Trillium amabile* Miyabe & Tatew. | B | Hidehiko Kamizono 3 | TI | KM242731 | KM242962 | KM242800 | KM242882 | - |
| Parideae | *Trillium angustipetalum* (Torr.) J. D. Freeman | A | F.H.Utech 84-008 | TI | KM242732 | KM242963 | KM242801 | KM242883 | - |
| Parideae | *Trillium camschatcense* Ker Gawl. | B | J.K.Hong, 042 | GCU | KM242733 | JN417473 | JN417383 | KM242884 | - |
| Parideae | *Trillium catesbaei* Elliott | A | H.Koyama *& al.* 6549 | TI | - | KM242964 | KM242802 | KM242885 | - |
| Parideae | *Trillium cernuum* L. | A | ND |  | - | KF613056 | AB017380 | - | - |
| Parideae | *Trillium chloropetalum* (Torr.) Howell | A | Chase 29048 | K | KM242734 | KM242965 | KM242803 | - | - |
| Parideae | *Trillium discolor* Hook. | A | D.E.Boufford 22828 | TI | KM242735 | KM242966 | KM242804 | KM242886 | - |
| Parideae | *Trillium erectum* L. | A | S.C.Kim 2012-003 | GCU | KM242736 | KM242967 | KM242805 | KM242887 | - |
| Parideae | *Trillium flexipes* Raf. | A | D.E.Boufford 18077 | TI | KM242738 | KM242969 | KM242807 | KM242889 | - |
| Parideae | *Trillium govanianum* Wall. ex D.Don | B | Chase 16955 | K | KM242739 | KM242970 | KM242808 | KM242890 | - |
| Parideae | *Trillium grandiflorum* (Michx.) Salisb. | A | S.C.Kim 2012-002 | GCU | KM242740 | KM242971 | KM242809 | KM242891 | - |
| Parideae | *Trillium kurabayashii* J.D.Freeman | A | F.H.Utech 84-382 | TI | KM242741 | KM242972 | KM242810 | KM242892 | - |
| Parideae | *Trillium lancifolium* Raf. | A | S.Farmer 20000117 | GCU | KM242742 | - | AB017394 | - | - |
| Parideae | *Trillium luteum* (Muhl.) Harb. | A | F.H.Utech 90-133 | TI | KM242743 | KM242973 | KM242811 | KM242893 | - |
| Parideae | *Trillium maculatum* Raf. | A | S.Farmer 19990006 | GCU | KM242744 | KM242974 | KM242812 | KM242894 | NC027738 |
| Parideae | *Trillium ovatum* Pursh | A | Chase 18989 | K | KM242745 | KM242975 | KM242813 | KM242895 | - |
| Parideae | *Trillium petiolatum* Pursh | A | F.H.Utech 84-243 | TI | KM242746 | KM242976 | KM242814 | KM242896 | - |
| Parideae | *Trillium recurvatum* L.C.Beck | A | F.H.Utech 87-034 | TI | KM242747 | KM242977 | KM242815 | KM242897 | - |
| Parideae | *Trillium reliquum* J.D.Freeman | A | F.H.Utech 83-135 | TI | KM242748 | KM242978 | KM242816 | KM242898 | - |
| Parideae | *Trillium sessile* L. | A | Chase 29047 | K | KM242749 | KM242979 | KM242817 | KM242899 | - |
| Parideae | *Trillium simile* Gleason | A | S.Farmer 20050171 | GCU | KM242750 | - | AB017407 | - | - |
| Parideae | *Trillium smallii* Maxim. | B | T.Kobayashi 44149 | TI | KM242751 | KM242980 | KM242818 | KM242900 | - |
| Parideae | *Trillium sulcatum* T.S.Patrick | A | S.Farmer 19920109 | GCU | KM242752 | - | AB017407 | - | - |
| Parideae | *Trillium tschonoskii* Maxim. | B | J.K.Hong, 037 | GCU | KM242753 | JN417474 | JN417384 | KM242901 | - |
| Parideae | *Trillium undulatum* Willd. | A | W.G.Dore 14157 | NY | KR233264 | KM242981 | KR233273 | KM242902 | - |
| Parideae | *Trillium vaseyi* Harb. | A | D.E.Boufford 23596 | TI | KR233265 | KM242982 | KM242819 | KM242903 | - |
| Xerophylleae | *Xerophyllum asphodeloides* (L.) Nutt. | A | R.L.Wilbur 7008 | NY | - | KM242998 | KM242834 | KM242924 | - |
| Xerophylleae | *Xerophyllum tenax* (Pursh) Nutt. | A | Chase 527 | K | KM242764 | JN417485 | JN417394 | KM242925 | NC027158 |
| **Liliaceae** | *Clintonia udensis* Trautv. & C.A.Mey. |  | J.K.Hong 053 | GCU | KM242695 | JN417446 | JN417357 | KM242840 | - |
|  | *Lilium hansonii* Leichtlin ex D.D.T.Moor*e* |  | D.K.Kim 05-026 | GCU | JX903929 | JX903239 | JX903655 | JX903510 | - |
|  | *Lilium longifolium* Griff. |  | ND |  | - | - | - | - | KC968977 |
|  | *Tricyrtis macropoda* Miq. |  | J.K.Hong 048 | GCU | KM242729 | JN417449 | JN417360 | KM242880 | - |
|  | *Tulipa uniflora* (L.) Besser ex Baker |  | M.W. Chase 751 | K | KM085921 | KM085540 | KM085669 | KM085795 | - |
|  | *Gagea lutea* (L.) Ker Gawl. |  | J.K. Hong 001 | GCU | KM085877 | AB034752 | AB024389 | KM085751 | - |
|  | *Calochortus uniflorus* Hook. & Arn. |  | M.W.Chase 13354 | K | KM085946 | KM085565 | KM085695 | KM085816 | - |
| **Colchicaceae** | *Colchicum speciosum* Steven |  | LHMS 266 | GCU | KC796810 | KC796866 | KC796916 | KM242841 | - |
|  | *Colchicum lineare* L. |  | ND |  | - | - | - | - | NC026785 |
|  | *Disporum smilacinum* A.Gra*y* |  | J.K.Hong 022 | GCU | KC796814 | JX903236 | JN417396 | - | - |
|  | *Uvularia perfoliata* L. |  | A.Adamsons 6154 | GCU | KC796829 | KC796881 | KC796932 | KM242905 | - |
|  | *Wurmbea dioica* subsp. *brevifolia* R.J.Bates |  | MEL 2301322 | GCU | KC796834 | KC796887 | KC796834 | KM242923 | - |
|  | *Gloriosa superba* L. |  | ND |  | KC796817 | D28867 | KC796920 | EU044626 | - |
|  | *Burchardia rosea* Keighery |  | Chase 2224 | K | KC796807 | KC796863 | KC796914 | KJ200328 | - |
| **Petermanniaceae** | *Petermannia cirrosa* F.Muell. |  | ND |  | AY465558 | AY465714 | JQ435572 | AY465662 | KU309331 |
| **Smilacaceae** | *Smilax rotundifolia* L. |  | ND |  | AY465554 | AY465710 | JQ711017 | AY465659 | - |
|  | *Smilax hispida* Raf. |  | ND |  | - | KF724360 | JF956414 | KF818423 | - |
|  | *Smilax china* L. |  | ND |  | - | - | - | - | HM536959 |
|  | *Heterosmilax japonica* Kunth |  | ND |  | - | JF941918 | JF954027 | KF818401 | - |
|  | *Heterosmilax chinensis* F. T. Wang |  | ND |  | - | FN870844 | JF954022 | KF818400 | - |
| **Campynemataceae** | *Campynema lineare* Labill. |  | ND |  | NC026785 | NC026785 | NC026785 | NC026785 | NC026785 |
|  | *Campynemanthe viridiflora* Baill. |  | ND |  | - | JN417506 | KM822196 | - | - |
| **Rhipogonaceae** | *Rhipogonum papuanum* C. T. White |  | ND |  | KC796847 | KC796898 | KC796951 | - | - |
|  | *Rhipogonum elseyanum* F. Muell. |  | ND |  | GQ497654 | GQ497672 | JN417372 | KC899417 | - |
| **Alstroemeriaceae** | *Alstroemeria pulchella* L. f. |  | ND |  | - | JX418007 | JX418009 | JX418005 | - |
|  | *Bomarea formosissima* (Ruiz & Pav.) Herb. |  | ND |  | - | JQ404724 | JQ404837 | JQ404601 | - |
| **Philesiaceae** | *Lapageria rosea* Ruiz & Pav. |  | M.W. Chase 17824 | K | KM198736 | KM198698 | KM198708 | KM198718 | - |
| **Philesiaceae** | *Philesia magellanica* J.F.Gmel. |  | ND |  | HQ901512 | HQ901562 | KC511342 | HQ901539 | - |
| **Luzuriagaceae** | *Luzuriaga marginata* |  | ND |  | - | JQ404757 | JQ404878 | JQ404650 | - |
|  | *Luzuriaga parviflora* |  | ND |  | - | AF307925 | JQ404883 | JQ404655 | - |
|  | *Luzuriaga radicans* Ruiz & Pav. |  | ND |  | NC025333 | NC025333 | NC025333 | NC025333 | - |
|  | *Drymophila moorei* Baker |  | ND |  | JX903931 | JX903241 | JN417413 | JX903512 | - |
| **Asparagaceae** | *Yucca glauca* Nutt. |  | ND |  | AY147637 | AY149380 | - | AF547014 | - |
|  | *Yucca queretaroensis* Piña Luján |  | ND |  | JX903739 | JX903135 | JX903546 | JX903320 | - |
|  | *Yucca schidigera* Ortgies |  | ND |  | - | - | - | - | NC032714 |
| **Amaryllidaceae** | *Agapanthus africanus* (L.) Hoffmanns. |  | ND |  | JX903728 | JQ273888 | JQ276383 | AF508405 | - |
|  | *Agapanthus coddii* F.M. Leight |  | ND |  | - | - | - | - | NC035971 |
| **Orchidaceae** | *Apostasia wallichii* R. Br. |  | ND |  | JX903906 | HQ182416 | JX903642 | JX903489 | - |
